# HIV prevention clinical trials’ community engagement guidelines: inequality, and ethical conflicts

**DOI:** 10.1080/11287462.2020.1773061

**Published:** 2020-06-05

**Authors:** Morenike O. Folayan, Kristin Peterson

**Affiliations:** a New HIV Vaccine and Microbicide Advocacy Society, Nigeria; b Department of Child Dental Health, Obafemi Awolowo University, Ile-Ife, Nigeria; c Department of Anthropology, University of California, Irvine, USA

**Keywords:** HIV, ethics, Cambodia, Cameroon, Malawi, Nigeria, PrEP, HIV prevention, clinical trials, community engagement

## Abstract

In 2004 and 2005, the first clinical trials were launched to investigate the use of tenofovir for HIV prevention in Cambodia, Cameroon, Nigeria and Thailand. Controversies erupted over the ethical integrity of the research protocol. We reflect on the events that led to the controversies and identified that scientific and ethical concerns raised by members of local communities at each of these sites were erased by trialists, causing crisis that led to premature shut down the early PrEP trials. In the aftermath of these trials, the World Health Organisation, UNAIDS, and AVAC developed ethics guidelines intended to recognize the concerns as authentic, and developed guidelines to improve researchers’ engagement of communities in biomedical HIV prevention trial design and implementation. Our findings suggest that the ethics guidelines are limited in its ability to address power inequalities that leads to voice erasures and non-recognition of local competencies. Rather the ethical documents enabled trialists to gain a new sense of authority through the interpretations of ethical research conduct enabling trialists regain power that can further entrench inequality and voice erasures. To address concerns with what seems an intractable problem, we suggested models of engagement for off-shored research may be the option.

## Introduction

In 2004 and 2005, the very first biomedical HIV prevention clinical trials investigating the use of an antiretroviral pill per day to prevent HIV infection – pre-exposure prophylaxis (PrEP) – were launched in Cambodia, Cameroon, Nigeria and Thailand (Ukpong and Peterson, 2009). These early PrEP trials were organized and funded by institutions in the United States including the National Institutes of Health, the Center for Disease Control, Family Health International, and the Gates Foundation; Gilead Sciences supplied the study drug [tenofovir]. Tenovir was an already existing marketed antiretroviral used as part of the second line treatment cocktail for HIV infection in the trial countries. Researchers recruited hundreds of study participants who were at high risk for HIV infection – female sex workers in Cambodia, Cameroon, Nigeria; and people who inject drugs in Thailand.^1^ Study participants were either enrolled into the active study arm where they were required to use tenofovir tablets daily, or they were enrolled in the control arm where they were required to use a placebo. All study participants were provided risk reduction counseling and male condoms (Peterson et al., 2007).

These trials encountered a number of controversies and debates in the host countries due to unethical trial design and study implementation procedures documented by HIV advocates (Ukpong & Peterson, 2009). These concerns ranged from calls for revision of the informed consent process, improved standards of prevention for study participants and standard of care for those who HIV sero-convert – those whose HIV status changes from negative to positive – during the trial. Other concerns included access to treatment of trial related injuries. These concerns are discussed in our analysis of the controversies.

As the trials were underway, these concerns went unaddressed, which led to local and international protests by HIV advocates and premature closure of the trials in Cambodia and Cameroon (Cooper, 2013; Forbes & Sanushka, 2009; IAS, 2005; Michael & Rosengarten, 2013; Sandy, 2012; Peterson et al., 2015; Ukpong & Peterson, 2009). The reason given by the trial sponsor (Family Health International) for the shutdown of the study in Nigeria was related to concerns about the local research site’s poor adherence to good clinical practice. The Thailand trial did not close prematurely but the protocol had to be amended to include access to harm reduction programs for study participants. Although a site in Malawi was originally scheduled, the country’s national ethics committee did not award Institutional Review Board’s approval (Peterson et al., 2015; Ukpong & Peterson, 2009).

After the trials closed, the International AIDS Society [IAS], Joint United Nations Program on HIV/AIDS [UNAIDS], and the Gates Foundation hosted several consultations with stakeholders, including donors, bioethicists, researchers, scientists, community activists and advocates, to examine what went wrong. These actors also considered how to continue biomedical HIV prevention research in the face of the controversies (Collins, 2005; International AIDS Society, 2005; Mellors, 2005; UNAIDS, 2006). The consultative meetings led to the development of ethics guidance documents that focused on ethical conduct of biomedical HIV prevention clinical trials. These include the “Ethical Considerations for the Conduct of Biomedical HIV Prevention Trials,” developed by the World Health Organization (WHO) and UNAIDS, which declared “stakeholder [trial participants and their community-based ally and advocate organizations] engagement” in biomedical HIV prevention trials an ethical imperative and a right (Slack et al., 2018; UNAIDS and WHO, 2007, 2012). WHO and UNAIDS agreed with the position of community advocates and activists in the guidance documents developed (Global Campaign for Microbicide, 2005, 2009; HANC, 2014; Miller et al., 2010). UNAIDS and the AIDS Vaccine Advocacy Coalition (now called AVAC) then developed the first comprehensive document on how to engage stakeholders in the research lifecycle, named the “Good Participatory Practice [GPP] Guidelines” (UNAIDS, 2012; 2011).

Those civil society organizations involved with the PrEP controversies also developed locally relevant documents. For example in Cambodia, a code of practice for researchers working with sex workers was created (Asian Pacific Sex Workers Network, 2007). In Cameroon, an inter-associative working group that addresses the protection of people who participate in research was established. The group works with agencies conducting research in Cameroon to facilitate community review of protocols prior to submission to ethics review committees (Réseau Ethique Droit et Sida, no date). In Nigeria, a consensus document on the standard of care for HIV prevention technology research was also developed (Folayan et al., 2011). In Thailand, the Thai AIDS Treatment Action Group [TTAG] developed the document recommending good participatory practices in biomedical HIV prevention trials in Thailand (TTAG, 2012). The HIV Prevention Trial Network, funded by the National Institute of Health, revised its ethics guidance document to reflect its expectations on community engagement with their clinical trials (HPTN, 2009).

The 2004–2005 oral tenofovir controversies revolutionalized the field of biomedical HIV prevention research in many ways. The most significant was that for the first time, the definition of clinical ethics extended beyond clinical researcher-trial participant interactions to include stakeholders like community representatives advocating on behalf of trial participant welfare. Secondly, the GPP formally defined the way community engagement in research should be conducted in an attempt to reduce the practice of tokenistic engagements: it provided researchers with guidelines on how to engage communities in trial design, trial implementation, and the dissemination of research results. It also provided stakeholders with an instrument to quantify and measure stakeholder engagement practices.

While these developments are welcome, the documents did not acknowledge how economic, social and geopolitical power differences impact community-researcher engagement. These power differentials are particularly problematic in off-shored research (Fairhead et al., 2006). This article discusses the early PrEP trial controversies in detail, evaluate actions taken to resolve the controversies, particularly the development of ethical guidelines documents, and discusses the limitations of these guidelines in resolving or helping to manage power dynamics associated with implementing off-shored HIV prevention research that leads to voice erasure. In this context, we identify voice as a distinct marker of expression of a collective through which they socially interact and self-identify. It is a unitary identity in the production of communication (Strine, 1997). We highlight how communities critically interrogated the authorizing assumptions about PrEP clinical trials, how the narratives of researchers “erased” these voices in public discussions, and how voice erasure can continue to happen in research-researcher relationships even though ethical guidance documents exists. We then conclude by making recommendations on how off-shored research can be administered to reduce the tensions that arise from the power differences in the absence of mechanisms that can build true partnerships between HIV prevention researchers and trial communities.

## Methods

We conducted 62 ethnographic interviews between 2005 and 2011.^2^ We also conducted participant observation in the early PrEP dialogues as they were unfolding at each of the sites. We interviewed a wide range of purposefully selected local and international key players. These included advocates such as AIDS activists, community representatives, trial participants, and basic, clinical and social science researchers. Also interviewed were research site staff, university ethicists and policy makers. These actors were selected based upon their involvement with the research design and implementation of the PrEP trials. Our interviews of research site staff included principal investigators, field workers and study coordinators. Interviews were conducted in privacy, audio-recorded and transcribed. Interviews were conducted face-to-face and lasted between 15 min and two hours. The interviews were semi-structured and conducted by either or both of the authors. Often, we offered participants refreshments before starting the interview to help create a relaxed environment. All interviewees were selected by snowball sampling techniques or were those with whom we had built rapport during the years we researched this study.

At international AIDS conferences and HIV research meetings, we conducted participant observations and interviewed members of international HIV prevention consortiums as well as feminist activists who first coined the term, “microbicide” – compounds used in the vagina or rectum that were tested for HIV prevention mostly during the 2000s. We interviewed scientists who conducted preclinical PrEP research in the 1990s. We followed and analyzed extensive debates on microbicide and PrEP trials taking place on African listservs (Journalists against AIDS and the Nigeria HIV Vaccine and Microbicide Advocacy Group listservs between 2004 and 2005).

We also reviewed and analyzed anecdoctal reports of the controversies that occurred in Cameroon, Cambodia, Nigeria, Malawi and Thailand; the literature that discussed the PrEP trial controversies^3^; documents that addresses ethical consideration in the design and implementation of biomedical HIV prevention trials (Miller et al., 2010; Selvin et al., 2008; UNAIDS and WHO, 2007); and accessible conference abstracts that discussed these controversies (Chigwedere et al., 2010; Peterson & Ukpong, 2008, 2013; Ukpong et al., 2008; Ukpong et al., 2010; Ukpong & Peterson, 2009). We also studied international ethics guidelines: UNAIDS/WHO (2007, 2012) Ethical Considerations in Biomedical HIV Prevention Trials, UNAIDS/AVAC GPP (2007, 2011) Good Participatory Practice Guidelines for Biomedical HIV Prevention Trials, and the CIOMS (2016) International Ethical Guidelines for Health Related Research Involving Humans.

Our approach to data analysis was that of “reﬂexive ethnography” wherein we drew on our data and research experiences as participant-observers as the tenofovir controversy in Nigeria and beyond, to interpret the dynamics and interactions that took place during the controversies. Ethnography and the use of reflection are well suited for this study because of our active engagement in the life history of the events we examined (Carpenter-Song & Whitley, 2013). We used “reﬂexive ethnography” as a social critique tool to examine the power imbalances produced through different social positions held by researchers and local communities. We discussed and reached consensus on the themes we identified as emerging from interview transcripts as well as findings from reports, literatures, conference abstracts, mentioned above. When we reached consensus on themes, we then conducted more in-depth review of the data to identify new emerging sub-themes, which we highlight in our findings.

Our use of reflexive-ethnography as our analytical research tool is appropriate for the study of research subject – power dynamics (Finlay, 2002). The approach requires that we as researchers, consider how a research process is structured around issues of power and dominance (Burman, 1990). It is a tool that helps to develop the processes and experiences that occurred during the research with the aim of developing a new construction of past reality that can be further interrogated (Riley et al., 2003). We recognize that researchers’ perspectives can shape reflections (Gill, 1998; Greed, 1990) but we limited the potential for this by contextualizing our analysis within the findings of other reports on the subject matter. This approach is an adapted social constructionist approach that argues for the interrogation of reflexive accounts.

## Results

### The community-based organizations involved with the early PrEP trials

The term “community” includes journalists, policy makers, social workers, clinical ethicists, bench scientists as well as community-based or non-governmental organizations linked to trial participants and involved in the PrEP debates. These actors considered themselves as part of an advocacy community, each playing different and collaborative roles. Members of these organizations who participated in the tenofovir debates possess a range of expertise including knowledge and competency to advocate for people living with HIV, sex workers, and people who inject drugs. Those community members who are ethicists and research scientists have additional competency to evaluate the ethics and scientific rationales of the trials. Local organizations had been advocating for HIV prevention in their geographical locations prior to the commencement of these HIV prevention trials. The organizations were:

- Woman’s Network for Unity, a national union of Cambodian sex workers founded in 2000;
- Réseau Ethique Droit et Sida (REDS) in Cameroon, an HIV research ethics advocacy organization founded in 1998;
- The New HIV Vaccine and Microbicide Advocacy Society in Nigeria (NHVMAS), an HIV prevention advocacy group comprised of research scientists, journalists, bioethicists, and AIDS advocates, founded in 2003;
- The Thai Drug Users’ Network and Thai AIDS Treatment Action Group founded in 2002.

### Concerns raised by community-based organizations

Local organizations emphasized the need for the trials to be designed and implemented in ways that respect and acknowledge the existence of local health needs. Advocacy communities in Nigeria and Cameroon called for the informed consent process to be revised so to ensure study participants understood trial objectives as well as the risks and benefits associated with study participation. This call was made due to concerns about the risk for therapeutic misconception – the assumption that clinical research is a health program (Reynolds et al., 2013) – in low research literacy settings. We found that the vast majority of the study participants in Nigeria believed that the study drug would actually prevent HIV because the concept of placebo was foreign to them. Such a phenomenon has the capacity to increase study participants’ risk of HIV sero-conversion. Communities hosting the trials had sub-optimal access to clean needles (in Thailand) and used condoms sub-optimally at all other sites (Ukpong & Falobi, 2005). Advocates at all the study sites also requested plans be made to ensure study participants who sero-converted had access to HIV medicines whose supplies were guaranteed by the research team.^4^ At the time of the study, all sites had limited access to HIV medicines, clean needles and condoms. Stigma associated with HIV infection was high and the risk of dying from AIDS-related complications was very high. Guaranteed access to antiretrovirals was considered ethical as the success of the study was dependent upon enumerating HIV sero-conversions.

In Malawi and Nigeria, the possibility of sero-conversion could mean developing resistance to tenofovir – a concern raised by both advocates and research scientists. In each of these countries, tenofovir is the second (and last) line in national HIV treatment protocols. For the research host communities, any resistance to tenofovir implied that study participants would have no access to HIV treatment that can be provided through the national program (Peterson et al., 2015; Peterson & Folayan, 2019; Ukpong & Falobi, 2005). Scientists, ethicists, and advocates in Nigeria as well as advocates in Cambodia also raised concerns that the study did not provide participants access to treatment of trial related injuries. Of concern were renal dysfunction and reduced bone mineral density associated with use of tenofovir (Barditch-Crovo et al., 2001). Moreover, at that time, little was known about complications associated with tenofovir use by HIV negative individuals (Thompson, 2005).

Finally, there were concerns with the standard of prevention. Advocates in Nigeria and Cameroon demanded that female sex workers be provided with and trained on the use of female condoms as part of the HIV prevention tools available to trial participants. In Thailand, community advocates requested for a needle exchange program and access to methadone for people who inject drugs enrolled for the study because only condoms were provided, which is not the most appropriate HIV risk reduction strategy for people who inject drugs.

### Actions that trial communities took to alleviate concerns found in the protocol

We found that local organizations attempted multiple times (using multiple media) to share their concerns about the trial protocol with the trial organizers. They sought opportunities to hold face-to-face meetings, wrote letters and emails to communicate grievances, and engaged in discussions over national listservs (Peterson et al., 2015; Peterson & Folayan, 2019). Some challenges included the failure to meet face-to-face with research team members; or when meetings did occur, their concerns about the trial design and safety provisions did not result in the revision of the trial protocol. Specifically, the PrEP trialists failed to either respond (Nigeria) or they provided negative feedback (Cambodia, Cameroon and Thailand) on suggestions for protocol revisions. Researchers in Cambodia and Nigeria directly informed community organizations that the issues raised could not be addressed; and in Cambodia, community advocates were informed that their request for medical care insurance for trial-related injury could not be granted (Sandy, 2012).

The organizations then turned to their long-term partners and allies within the local and international AIDS community, seeking information to help improve their advocacy efforts while trying to negotiate with researchers for improved research implementation practices. In Cambodia, the Women’s Network for Unity consulted with the Women’s Agenda for Change in the United States. In Cameroon, REDS consulted with Act-Up Paris, Medecins Sans Frontières in Cameroon, the Cameroonian Red Cross, and the Society for Women and AIDS in Africa [SWAA], Cameroon. NHVMAS in Nigeria reached out to Journalists against AIDS in Nigeria, colleagues at SIDACTION in France, and REDS in Cameroon. TTAG in Thailand reached out to Medecins Sans Frontières in Belgium (Ukpong and Peterson, 2009). Swift and prompt communication among these organizations was a result of long-term collaborative AIDS activities and friendship that existed long before the 2004 PrEP trials commenced.

The Cambodian and Cameroonian organizations held press conferences (Ukpong & Peterson, 2009; Yomgne, 2009), and public demonstrations were conducted to draw attention to the Cambodia and Cameroonian sites (Ukpong & Peterson, 2009; WNU, 2004a, 2004b; Yomgne, 2009);^5^ advocates in Thailand published open letters in the Lancet and Plos One journals (Chua et al., 2005; Jintakarnon et al., 2005); and in Nigeria there were published newsletters as well as an extended discussion that took place on a national HIV/AIDS listserv (Peterson & Folayan, 2019; Ukpong, 2009; Ukpong & Falobi, 2005).

Prior narratives about these controversies indicated that AIDS activist organizations located in the Global North opposed the PrEP trials and controlled the opinion of advocates at the trial sites (Forbes & Sanushka, 2009). This assumption that only AIDS activists in the Global North could steer trial opposition erases the competency of local scientists, ethicists, and organizations to speak for themselves. Trialists controlled this discourse in the media and in international medical journals without offering any reasonable proof of such assertions (Grant et al., 2005; Lange, 2005). In the years following the early PrEP controversies, we found that this discourse turned into “common knowledge” at AIDS conferences.

We perceived that the early PrEP trialists were not aware that local organizations had the ability to mobilize international voices to amplify their concerns. Trialists insisted that international actors drove local demands – we witnessed many trialists assert that trial communities were not competent enough to evaluate protocols and lodge complaints. ActUP Paris was especially singled out for the trial closures . The idea that local communities in the global south cannot think critically about the science and ethics of research, and organize themselves for action, is an erasure of local community competency. Worse still, the actions by local communities for self-preservations were labeled as a distraction to the pace of science (Rennie et al., 2010).

### Scientific and ethical merits of raised concerns

Community concerns were initially rebuffed by trialists. Concerns pertaining to the science and ethical validity of the PrEP trials were given due consideration in ethics guidance documents developed by research organizations such as the Centers for Disease Control and Prevention (2011), Family Health International (Macqueen et al., 2012), National Institute of Health (2014) and amfAR et al (2015). The notion that HIV expertise is only possessed by the trialists raised questions of power in terms of who had access to media, access to publication, and who had the power to control the narrative on “what happened” at the host sites. Silencing community concerns led to limited discussions, until a crisis ensued. After the controversies, changes made to PrEP trial design and implementation – and by extension, its applicability to other types of biomedical HIV prevention clinical trials – showed that many of the scientific and ethical concerns raised by local communities about the trial were valid. Changes made on scientific and ethical grounds include:

- *New Standard HIV Prevention Packages.* Guidance point #13 of the UNAIDS/WHO document (2012, 2007) provided that people who inject drugs (PWID) are now entitled to clean needles and syringes through needle exchange programs. At the Thailand PrEP site, PWID only had access to condoms (Kaplan, 2009). Female condoms for trials recruiting female sex workers are now also consider part of a standard prevention package, which were unavailable during the PrEP trials (Ukpong and Peterson, 2009). Moreover, male and male to female transgender circumcision was included as a standard HIV prevention package for the STEP/Phambili HIV vaccine trial; and the trial design was modified when there was evidence that PrEP was effective (Janes et al., 2013).
- *Facilitated access to HIV management*. Potential study participants can sometimes be denied trial participation because they discover that they are HIV positive upon screening. They could also seroconvert during the trial. The 2004 PrEP research protocol required that persons who were HIV positive be referred for treatment without due consideration for how access would be possible. The ethics guidance documents now recommend that before a trial commences, research teams should directly arrange HIV treatment access for those who screen out or seroconvert. (Section 3.11 UNAIDS/AVAC, 2011; Guidance Point 14 UNAIDS/WHO, 2007).

### Unresolved concerns and entrenched inequalities

Though the ethics documents developed in the wake of the early PrEP trial controversies resulted in changes in some clinical practices as specified above, there were at least three other issues that went unresolved. The first was that drug safety profiles were unknown for the trial population. The trial was technically a Phase 2b, referred to as a “pivotal trial”. However, advocates claimed that a Phase 2b trial was inappropriate because there were no substantive preclinical and Phase I trial data that evaluated the safety of tenofovir for use as PrEP. It also queried the conduct of a trial using systemic antiretroviral (in the form of pills) for a population that were poorly adherent to the use as exemplified by pills for contraception. They also advocated for a PrEP trial to evaluate safety in a less health vulnerable population first. Second, scientists in Nigeria argued that malaria co-infection should be monitored and understood in the context of PrEP. Nigeria is a malaria-endemic country and such research information would have been critical in their view. Recent evidence suggests that tenofovir-associated renal dysfunction may reduce chloroquine (a drug used for the treatment of malaria) clearance (Fehintola et al., 2011). Third, advocates in Nigeria also disagreed with the trial’s adverse events scale. This scale is a categorized breakdown of low to high health problems (adverse events) related and unrelated to the trial. It signals when a trial participant should withdraw from the study by defining specific adverse effects on a Grade 1–4 scale, Grade 4 being the worst health effects. The protocol mandated the following: the drug is withdrawn due to an unrelated event at Grade 3; when the participant reaches less than or equal to Grade 2 it is restarted. At Grade 4, the trial volunteer should be withdrawn permanently. Given the poor health profiles of the study population, NHVMAS argued that if a Grade 3 or 4 adverse event occurs, even if it is considered unrelated to the study drug, the drug should be discontinued (Peterson & Folayan, 2019). These unresolved concerns pertain specifically to the trial design driven by scientific rationales. Co-designing a clinical trial with local communities is not considered germane to community engagement by the major guidance documents.

## Discussion

Our review of the 2004–2005 tenofovir controversies highlights salient issues still relevant for planning and implementing offshored or locally designed HIV prevention trials. We found that community engagement is upheld as the primary, if not only, intervention that can alleviate trial tensions. Co-designing a trial between trialists and their counterparts in offshored settings is not considered part of community engagement, nor is it considered an important ethical approach to offshored research. Yet given the debates we have enumerated, co-designing a trial could have gone a long way in easing tensions and ensuring better trial success (West Slevin et al., 2008). Regardless of how fruitful community engagement might be, trialists’ total command of the trial design excludes local community input, which entrenches trialists’ decision-making power.

The power imbalance further enabled PrEP trialists erase voices of local communities’ by not given due consideration despite the considerable value of their concerns. Local communities’ capacity to organize themselves as well as their ability to raise valid scientific and ethical concerns about research protocols were not recognize. This attempt to erase the voices of the local communities, eventually caused significant disruptions of the trials.

There had been past recognition of the potential for the North Atlantic researchers to inadvertently exploit resource-limited settings (Benatar, 2000). Our research has identified that the power imbalance that had been observed in the North–South collaborative research can also result in the erasure of voices that can otherwise, contribute meaningfully to equitable design and implementation of non-exploitative o research. For example, in this study, we identified that community advocates called for the institution of standards for prevention and standards of care for trial participants that can reduce the risk for contracting HIV infection more suitable for their needs, and the risk for trial related injuries that can hamper their ability to be bread-winners post trial. The validity of these conversations by locals in Cambodia, Cameroon, Nigeria and Thailand were undermined by ascribing the agitation for protocol review as resulting from poor research literacy that can be resolved by research education for the study community by researchers. In addition, the local voices were erased through its misrepresentation as the voices of Global North activists (Forbes & Sanushka, 2009). The contributions of the local voices to the design and implementation of the early PrEP trial designs were only heard and authenticated following protests, disruptions of the trials by local and international activists, and mediation by international agencies (UNAIDS, 2006) despite several calls by the community activists at the study sites in Africa and Asia for protocol amendment to address their concerns (Ukpong & Peterson, 2009). Ethical guidelines developed in the wake of the PrEP controversies represents further attempts to ameliorate the tensions that could arise from community interactions with clinical trialists. Sadly, these local voices receive no credit for their contributions to improving the ethical conduct of biomedical HIV prevention research.

There had been no discussion on the potential for this erasure prior to now. The ability for those with power to erase voices of the less powerful have significant implications for undermining efforts to promote equitable engagements between researchers and the less powerful community they engage with. While the structural context that creates the power imbalance persists, equitable relationships in these situations will be dependent on individual goodwill, which is a very unstable phenomenon (Nelson, 1953). The development of ethical guidelines is an attempt to address the tension resulting from the power imbalance (Parker & Kingori, 2016; Varga-Dobai, 2012). In reality, the guidelines transformed the interaction between researchers and the research community into a structured collaboration that can be monitored through checking off a list of activities. Sadly, this transformation enabled ethics to gain a new sense of authority, mainly for researchers: they are now able to quantify interactions with communities and count them as evidence of community engagement. For example, the UNAIDS/AVAC, 2011 ethical guidelines were developed to improve collaboration, communication and efficient use of resources. In practice however, community engagement is employed to ensure the smooth implementation of off-shored research (MacQueen & Auerbach, 2018): it does not empower local communities to resolve the inequities of power relationships as off-shored research itself is dependent on inequality to function (Sunder Rajan, 2017).

Ethics guidelines inadvertently created an institutionalized divide between the community and the research team – local communities who often use participation in clinical trials to maximize their welfare while researchers portend to help the community by generating trial evidence (Varga-Dobai, 2012). The divide once again arrogates power to researchers who are assumed to have the expertise for research protocol development; and obscures inequality by negotiating space for local communities to be at the table to discuss research protocol development. This dichotomy gives trialists power to command the definition of ethics and good practices and entrenches the structural determinant of inequity it tries to address. That is, where the decision on local communities’ engagement is abrogated to trialists, and the ability to exert power is retained in a new disguise of ethical compliance. There is currently no evidence to suggest that the multiple guidelines developed as a systems approach to resolve the power differences in North–South collaborations (Inter Academy Council, 2012; Krubiner & Hyder, 2014; Thompson, 2013) can resolve the structural problem of power inequities in clinical research (Ammann, 2016).

Without resolving the challenges associated with power inequality in researcher-community relationships in off-shored research, vulnerable local communities will continue to bear the brunt, which leads to exclusion from and or limited freedom of participation, and the emergences of new systems of repression when local communities assert their voices (Permalink, 2018). Researchers are powerful no matter the context from which they operate (Riley et al., 2003).

One way out may be to consider participation in clinical research as a social contract that can be negotiated when a trial is being conceptualized, one that may enable both parties to draw up mutual terms of engagement for co-created trial designs and research participation. Social contract and social contract theories have largely being applied to discuss the relationship between citizens and the state. We find the contractarian rather than the contractualist approach (Schaefer, 2019) suitable for guiding research-researcher relationships that is contractual in nature, with a similitude of a governance-governed relationship.

The contractarian social contract theories recognize the bias stakeholders bring to the table, and the disconnect between individual goal pursuit and social welfare (Oakeshott, 1962). It posits stakeholders are already moral agents replete with narrow and personal interests and biases; and recognizes that political and moral constraints enhances the non-moral interests of those who abide by them (Darwell, 2008). It also recognizes that contractual agreement are non-existence in situations like research-participants relationships, and as such a model that aids with identification of the set of constraints in a relationship that rational individuals should endorse, is needed (Schaefer, 2019). It proposes that parties need to bargain to gain acceptance; and that through cooperation and coordination, inconspicuously guided by moral or political rules that structure interpersonal interactions, human beings can improve their lot by furthering the personal ends of the bargain and abide by them (Buchanan, 1975; d’Agostino et al., 1996; Gaus & Thrasher, 2016; Schaefer, 2019).

The Council for International Organizations of Medical Sciences (CIOMS) guidelines for health related research involving human subjects had alluded in unclear terms, to social contracting model for research-participant engagement when it stated that: *“Researchers, sponsors, health authorities and relevant institutions should engage potential participants and communities in a meaningful participatory process that involves them in an early and sustained manner in the design, development, implementation, design of the informed consent process and monitoring of research, and in the dissemination of its results”* (CIOMS, 2016). The UNAIDS/WHO and the UNAIDS/AVAC guidelines on community engagement with biomedical HIV prevention research had also promoted consultations between researchers and participants. The documents had however, not been explicit in their request for a contractarian social contract thereby limiting commitments of researcher stakeholders to such obligations.

The effectiveness of a contractarian social contract is however limited by the need for formal or informal, external or internal rules and mechanisms of enforcement. For research, the formal enforcement of rules governing research has largely rested with research regulatory agencies like the Institutional Review Boards and drug regulatory agencies. The competency of research regulatory agencies in many resource limited is generally weak; with their ability to monitor research and their compliance with ethics guidance more so. This limitation can however be effectively addressed through capacity building and appropriate support (Bain et al., 2018).

The role of ethics committees to enforce social contracts can be complimented by the watchdog role played by advocates and community activists. These actors have played multiple successful roles as informal external or internal mechanism of enforcement of research related requirements as exemplified by their success with pushing the boundaries for compliance with moral obligations that promote people-centred ethics standards for biomedical HIV prevention research (Peterson & Folayan, 2019; Philpott et al., 2011).

The effectiveness of a contractarian social contract is also limited by the feasibility that the social contract agents must have similar evaluative standards (Gaus & Thrasher, 2016; Moehler, 2018); and the ability for group pressures to affect rationality through influence of beliefs and consequent behavior (Schaefer, 2019). In a relationship between culturally diverse persons with differing research literacy and competency, the prospect for similarity in evaluation standards at the commencement of the contractual relationships is low as individuals starts the relationship with certain prior beliefs. The tendency for rationale thinking increases as individuals update their prior beliefs based on new information they assimilate (Muldoon et al., 2014). Research relationships built on a social contract model will therefore require time to nurture; and ensured access of stakeholders to information that enables them make accurate predictions (Schaefer, 2019). Time and access to information however, does not rule out the probability for divergence of preferences in such consultative processes. Consensus on opinions will be reached over time and earlier in societies intrinsically guided by moral or political rules that structure interpersonal interactions and decision-making processes. The social contractual model is however, still not well developed as the proposed approaches have largely been limited to two-persons rather than groups, bargaining (Kalai & Smorodinsky, 1975; Nash, 1950; Rubinstein, 1982)

An alternative approach to the social contract model is the labor engagement model for operating research contractual agreements. This proposition recognizes the long traction in trying to resolve the research relationship dynamics associated with the power inequality between researchers in the global North and resource-poor study communities; and the low likelihood of resolving this power dynamics in the short and medium term (Schneider, 2017). Folayan and Allman (2011) had earlier proposed research participants get paid for services. They identified the pros and cons- of this model, and suggested it be regulated by the labor laws that governs wage payment to trial participants. Laws governing occupation safety (with research defined as an occupation, research enterprises identified as the employer of labor and study participants as the labor) may well be applicable to the enterprise. Country and international laws will define safety terms for study participants and regulate essential protections and economic benefits. This approach recognizes the role of international research to provide benefits and payments to participants in resource-limited setting as a means of meeting their micro-level ethical responsibilities and providing the means of addressing macro-level issues of social justice (Njue et al., 2014). Moreso, it helps to resolve the ethical debates and arguments on the role of payment for research participation (Grady, 2005; Permuth-Wey & Borenstein, 2009).

The wage-pay model and other mechanisms for paying research participation (market, reimbursement, appreciation) is already in practice albeit this practice is not formalized through a labor system. The wage-payment model pays more for more work and appears to us the most feasible working mechanism for remunerating study participants for their engagement with research (Brown et al., 2019). There are, however, many arguments against concepts that promote payment for research participation through any model for multiple reasons (Denny & Grady, 2007; Lynch et al., 2019; Macklin, 1981; Resnik, 2015) one of which is that participants become passive actors in the process. Thus, it is argued, they do not respect the principle of autonomy (Smids & Nyholm, 2019). We argue that it is a principle that has not been respected anyway because of the persistent nature of erasing local concerns in offshored research settings. The voices and interest of research participants can be heard through labor unions that are able to use their collective strength to bring fairness to the workplace (Clark & Sadler, 2010).

Negotiating contractual agreements governed by labor laws for the research enterprise will be froth with a new set of problems though not likely of the type and magnitude resulting from the ethical requirement of researchers’ bonded by moral duties not to exploit. One of such problems is the possibility of conducting research studies in resource-limited settings from which the local community and study participants may not benefit. The wage-pay model will likely limit the co-creation of trials. An adoption of the labor-wage model inadvertently implies the acceptance of the current relationship status between researcher-participants in resource limited setting and promotes monetary gains from the relationship. The labor laws are used as instruments to limit exploitation; and use labor systems to harness formal and informal, external and internal rules and mechanisms to enforce these laws.

These models while not perfect, can enhance adherence to ethical guidelines in meaningful ways. We have tried to highlight the need to shift from a dependency on ethics guidelines as a way to resolve the tensions that can arise from the power imbalance between researchers from the north and the community in the south, to identifying complimentary systems for implementing ethics guidelines that can address the imbalance. Effective systems needed to promote partnerships of researchers with the local community^6^ require that researchers identify local communities as *knowledge holders and producers*. It implies that researchers will need to accept a reduction in their power to define research processes. This however does not rule out the ability of the researcher to reclaim the position of power through the use of institutionalized authority (Riley et al., 2003). In this situation, adherence to the ethics guidelines allows researchers to hold a temporary position of being collaborative, but reclaims the power through the narration of the dialogues from their perspectives. These suggestions by no means address all the intractable and inherent problems of inequality in clinical research. They however, can at least slightly off-set assumptions that the development of ethical guidelines can truly address inequality.

In conclusion, we propose that research-community engagement in resource-limited settings should recognize global and politically designed intractable power differences. As such, it is necessary to enact new research-community engagement paradigms that rewards communities for their contribution to the advancing of pharmaceutical science that is mostly destined for drug markets in the global north. As long as the research regulatory systems fail to address power differences that lead to erasing community concerns, local communities will find ways to exert their own agenda onto the conduct of these trials – many of which are in conflict with the objectives of the clinical research.

## Data Availability

Transcripts are accessible from the authors on request.

## Notes

1 Another trial was planned and concluded by Family Health International (non FHI360) in Ghana. It was the only human efficacy trial of PrEP implemented and concluded before the iPrEx study. The result of the trial was reported by Peterson et al. at the16th International AIDS Conference, Toronto titled ‘Findings from a double-blind, randomized, placebo-controlled trial of tenofovir disoproxilfumarate (TDF) for prevention of HIV infection in women’. The team published the result of the study from the Ghana, Cameroon and Nigeria sites (Peterson et al, 2007). The result of the trial was insufficient to prove that tenofovir PrEP was effective at preventing HIV infection. Tenofovir use in HIV-negative people did not raise any severe safety concerns.

2 Ethics approval was obtained for the study from the University of California Irvine, and Institute of Public Health, Obafemi Awolowo University, Ile-Ife, Nigeria and the University of Malawi. Oral consent was received from study participants after the aim of the study was explained to them, risk and benefits associated with study participation was discussed, confidentiality was assured, and their freedom not to engage with the study was highlighted.

3 Some of the reference articles studies specific to the trials included Chua et al., 2005; Jintakarnon et al., 2005; Ukpong & Falobi, 2005; UNAIDS, 2006; Mills et al., 2005; Singh & Mills, 2005; Ukpong & Peterson, 2009; Forbes & Sanushka, 2009; Michael & Rosengarten, 2013.

4 At the time of the study, access to antiretroviral therapy was only feasible when the CD4 count was as low as 200/mm^3^. Since the CD4 count was expected to be high at the time of sero-conversion, the need for antiretroviral treatment would still be many years down the line. The demand was therefore the need to put in places mechanisms that would ensure study participants get access to these drugs when the need arise through the issuance of health insurance as demanded by sex worker in Cambodia; or the registration of HIV sero-converters into HIV treatment programs in Nigeria.

5 This included the now infamous demonstration at the Gilead Sciences booth at the 2004 International AIDS Conference held from 11 to 16 June in Bangkok.

6 A framework for Community Based Participatory Action Research was developed by a group of organizations and published in 2011. Information about how participatory approach to research can be facilitated is accessible at https://hc-v6-static.s3.amazonaws.com/media/resources/tmp/cbpar.pdf.

## Geolocation information

The review covered studies conducted in Cambodia, Cameroon, Malawi, Nigeria and Thailand.
